# No long-term influence of movement restriction regulations on the contact-structure between and within cattle holding types in the Netherlands

**DOI:** 10.1186/1746-6148-8-188

**Published:** 2012-10-11

**Authors:** Henriëtte Brouwer, Chris J M Bartels, Arjan Stegeman, Gerdien van Schaik

**Affiliations:** 1 Department of Diagnostics, Research & Epidemiology, GD Animal Health Service Ltd, Deventer, the Netherlands; 2 Faculty of Veterinary Medicine, Department of Farm Animal Health, Epidemiology of Infectious Diseases, Utrecht University, Utrecht, the Netherlands

## Abstract

**Background:**

More and more countries hold databases on cattle movements. The primary purpose of the registration of cattle movements is to provide data for quick tracing of contagious animals in case of disease outbreaks and food safety scares. Nevertheless, these data can also be used for analytical studies to get insight into the nature of the contact structure between and within cattle holding types. This paper focuses on the effect post-2001 FMD movement regulations have had on the number of cattle movements between different and within the same cattle holding types. Important characteristics and dynamics of cattle movement patterns of Dutch cattle holding types were identified using data on cattle movements after the 2001 FMD outbreak.

**Results:**

The results showed that in 2001, just after the FMD outbreak when strict movement restriction regulations were in force, a reduced number of cattle movements was seen compared to before the FMD outbreak. However, the number of cattle movements off-farm for live trade and the number of imported cattle increased in the period 2002–2004 to higher levels than expected, i.e. to levels almost as high as before the FMD outbreak, despite operative movement restriction regulations. As the number of cattle movements to and from traders strongly decreased just after the FMD outbreak in 2001, traders regained their central role again in the network in the years 2002–2004.

**Conclusions:**

Quantifying the Dutch cattle contact structure between and within holding types up to 3.5 years after the FMD outbreak gave evidence that the post-FMD movement restriction regulations were not able to reduce the number of cattle movements in the longer term. With that the risk of a large epidemic increased. Quantifying contact structures based on animal movement data between different and within the same cattle holding types is important for targeting disease control and for assessing compliance with legislation.

## Background

Contacts between farms via cattle movements are considered to be an important risk factor in the spread of infectious diseases within and between animal holdings. Movement control and tracing of infections are among the first actions taken by veterinary authorities during disease outbreaks. To enable such measures, a registration system of cattle movements is crucial. Consequently, many countries have established authorities to register cattle movements, examples are the Cattle Tracing System in the UK [1], the National Livestock Identification System in Australia [2], the Central Husbandry Register (CHR) and Central Cattle Register (CKR) in Denmark [3], the Livestock Ranch Official Certification Program in Chile [4] and the Identification and Registration System in the Netherlands [5]. Increasingly, the mandatory reporting of cattle movements between holdings are used by researchers for mathematical modelling to investigate the spread of for example food-and-mouth disease (FMD) [6-8] and scrapie [8]. In addition, in the UK and Denmark movement data have been used to describe contact structures between cattle, pig or sheep holdings [8-10] to define risk-potential networks. This provides valuable information on the possibilities of disease transmission in the period between introduction and detection of infection. In addition, the efficacy of movement restriction regulations can be determined in the short and long term [11,12].

After the end of the 2001 FMD outbreak in the Netherlands (on June 25^th^ 2001) some of the movement restriction regulations were retained to reduce the number of cattle movements and, consequently, decrease the risk of transmission of infectious diseases in the future. The first regulation prohibited the gathering of cloven-hoofed animals for a period shorter than 30 days [13]. The second regulation prohibited the movement of cloven-hoofed animals off-farm within 30 days after a cloven-hoofed animal had been moved on-farm [14]. In May 2003, these regulations were eased to 21 days for all holding types instead of 30 days. These regulations made trading cattle for farmers more restrictive and complex. An exception to this rule were the markets and so-called cattle collection centres, where cattle were gathered for distribution to Dutch farmers or for export. These centres and markets were not allowed to gather cattle until June 2001. After June 2001, it was allowed to gather cattle in markets and collection centres on a daily basis given that strict hygiene protocols were met.

Velthuis and Mourits [11] used the I&R-database to compare the contact structure of the Dutch cattle industry before and immediately after the FMD outbreak. They concluded that due to the above mentioned regulations, the number of cattle movements off-farm for live trade had been reduced significantly in the year after the FMD outbreak and with a distinct change in the overall contact structure between and within holding types. Especially the number of cattle movements from dairy holdings to cattle collection centres was strongly decreased, whereas an increase in the number of cattle movements from dairy to beef holdings was seen. These changes were supposed to result in a reduction in size of epidemics. However, in the UK it was demonstrated that despite movement restriction regulations, the potential for large epidemics in the British cattle population has increased in the long term [12].

Due to differences in the cattle contact structure and regulations between countries, it was unknown how farmers outside the UK have adapted their cattle movements to threats imposed by highly contagious diseases. These differences could also have a different impact on holding owners“ responses to these movement restriction regulations. Because the European Union is a free trading zone, the national contact networks of the member states are interconnected. Therefore, it is important to quantify differences in contact structures between countries.

This paper focuses on the effect post-2001 FMD movement regulations have had on the number of cattle movements between different and within the same Dutch cattle holding types. This knowledge is important to determine the efficacy of movement restriction regulations in the short and longer term. In addition, it can be used to set up a country-specific targeted disease control. In the Netherlands, cattle herds are subdivided into six nationally defined holding types (Table 1). These holding types are driven by type-specific authorities and have to meet different disease control regulations. Because movement restriction regulations can have different consequences for different holding types, we wanted to know what the effect of these regulations were on the holding type level.

**Table 1:** Definitions of cattle holding types in the Netherlands

***Holding type***	***N***	***Definition***
Traders	374	Moving at least 20 animals on- and off-farm per year with a mean time present ≤ 60 days (including dealers, cattle shows, markets and cattle collection centres)
Young stock raising holdings	1,140	Holdings that raise young stock for dairy holdings
Beef holdings	3,283	Holdings keeping calves for veal production and/or bulls for bull meat production
Suckling cow holders	4,624	Holdings keeping suckling cows
Small-scale holdings	15,310	Holdings that have on average 20 animals or less present in a year
Dairy holdings	23,544	Holdings keeping dairy cows for milk production

## Methods

Since we only used anonymous data from existing databases no informed consent from the holding owners was required.

### Description of identification and registration (I&R) database

In the Netherlands, each cattle holding is obliged to report every cattle transport, birth or death to the national Identification and Registration (I&R) organisation. Each notification in the I&R-database consists of a unique farm identity number (UFI) related to one specific holding, an identification number of an animal, the birth date of the animal, the move on-farm code (birth, move on-farm, import) and move off-farm code (move off-farm for live trade, slaughter, death, export) and date of the movement. In this study, all cattle movement data from the Dutch I&R-organisation between July 2001-December 2004 were used.

### Applications of the I&R database

First, the I&R-database was used to define holding types and to distinguish different types of cattle movements. Second, cattle movements between cattle holdings after the FMD outbreak were determined and compared with cattle movements before the FMD outbreak [11] to explore if cattle holding owners' responses to movement restriction regulations were different in the longer term (2002–2004) as compared to the short term (July-December 2001). In addition, the number of cattle movements between and within holding types was quantified over time and network features were determined per holding type.

#### Definition of holding types

Each year a cattle holding was given one of six holding types (Table 1). The average number of animals present in one calender year was calculated and divided into three age classes (<1 year old, 1–2 years old and >2 years old) using the move on-farm, move off-farm and birth dates from the I&R-database. In addition, the number of births and the number of animals that moved on-farm and off-farm in a year with their mean time present on the farm were determined. Based on these data and the mean proportions of male and female animals present on the farm, pre-established definitions [11] were used to define the holding types.

#### Types of cattle movements

From the I&R-database five types of cattle movements were distinguished: 1) off-farm for live trade, 2) off-farm for slaughter, 3) off-farm to the rendering plant for destruction, 4) off-farm for export and 5) import.

An animal movement off-farm for live trade consisted of linking two notifications with the same unique animal identification number. Only movements for which the off-farm movement could be traced forward to an on-farm movement within a 14-days period were selected for the analyses [11]. Animals that were moved off-farm for slaughter, destruction or export or moved on-farm by import were defined as such in the database.

#### Cattle movements before and after the FMD outbreak

The mean number of cattle movements per month per year was determined for each movement type in the study period July 2001 to December 2004. In 2001, the after FMD period consisted of six months (July-December). Therefore, for each year only cattle movements in the period July-December were used to be able to compare cattle movements across years (i.e. to correct for seasonal effects). In addition, these numbers were compared with the mean number of cattle movements determined in the period before the FMD outbreak from July to December 2000 [11] to quantify the effect of the retained movement restriction regulations after the FMD outbreak. Differences in the mean number of cattle movements between years were tested, using a Kruskal-Wallis multiple comparison test (P ≤ 0.05).

#### Cattle movements between and within holding types

For the transmission of diseases within the Netherlands, cattle movements *off-farm for live trade* were considered as potentially risky contacts, in contrast to movements to slaughterhouses, to the rendering plant or abroad. The mean number of cattle movements off-farm for live trade per month was determined to quantify the contacts between and within Dutch cattle holding types for each year. For the identification of possible changes in the contact structure in the long term, for each year only cattle movements in the period July-December were used to be able to compare cattle movements off-farm for live trade across years.

The contact structure between and within holding types was visualized with the program UCInet (Analytic Technologies, Harvard, MA) for the years 2001 and 2004 to illustrate the change from 2001 to 2004. The data were represented as a network with six nodes (holding type) and edges representing the number of cattle movements between the nodes per year. Within node movements were represented by loops [15]. For a clear visual presentation of contact structures, only cattle movements between and within holding types with a minimum of an arbitrary number of 1,000 cattle movements per month were shown.

#### Network features

Many infectious diseases are introduced by moving infectious animals on-farm. Trading cattle, therefore, is a high risk factor for the transmission of diseases within the cattle contact structure. When movements involve long distances, diseases can easily spread to other areas. The following network features were determined per holding and aggregated per holding type:

The number of animals that move on-farm (i.e. open/closed farming system) in the last 12 months

Holding contacts:

1. The mean percentage of holdings with off-farm movements in a month

2. The number of different holdings an UFI has contact with (degree) per month

3. The number of contacts with the same holding per month

4. The number of animals moved per contact per month

Direct distances of cattle movements between holdings

The network features ‘mean percentage of holdings with off-farm movements', ‘holding contacts’ and ‘direct distances’ were based on *off-farm movements for live trade*, because these movements were considered as risky contacts.

##### Closed holding system

For each quarter the number of animals that were moved on-farm in the last 12 months was calculated. Based on this number, each UFI was set to an open (at least one animal moved on-farm in the last 12 months) or closed (no animals moved on-farm in the last 12 months) holding system per quarter (q1 = January-March; q2 = April-June; q3 = July-September; q4 = October-December). We could determine whether a holding was set to a closed holding system from July 2002 on, because on an earlier date the FMD period would be included in the definition for a closed holding system.

##### Holding contacts

**Figure 1:**
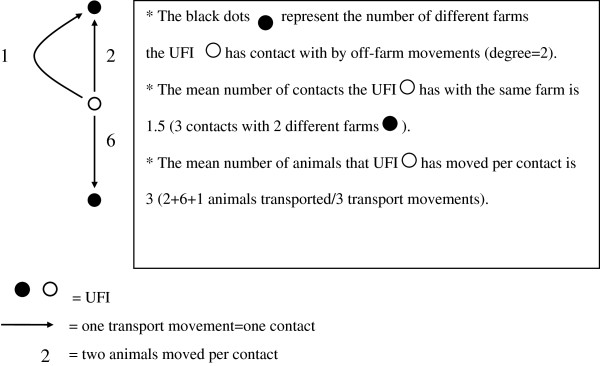
**Overview of the holding contacts.**.

For each holding type, the mean percentage of holdings with movements off-farm for live trade per month was determined. In addition, the number of different holdings an UFI has contact with (degree) was determined. Therefore, the mean degree per month was calculated over the period July 2001 to December 2004. Concurrently, for each holding type the mean number of contacts an UFI has with the same holding and the mean number of animals moved per contact were determined per month (Figure 1).

##### Calculation of the distance

For each holding type, the direct distance for all *movements off-farm for live trade* per month was estimated. Therefore, x and y co-ordinates for each UFI were used (source; GD, Animal Health Service Ltd., Deventer). If (x1, y1) are the co-ordinates of the UFI moving animals off-farm and (x2, y2) are the co-ordinates of the UFI moving animals on-farm, the Euclidean distance between two holdings was calculated. To compare Euclidean distances between holding types, these distances were divided in two categories: 0–40 km and >40 km.

## Results

### Cattle movements before and after the FMD outbreak

In 2001, in the six months after the FMD outbreak, there was a significantly lower number of cattle movements off-farm for live trade compared with before the FMD outbreak (2000), but despite the movement restriction regulations the numbers increased in the subsequent years to levels that were not significantly different from before the FMD outbreak (Table 2). In addition, the import of cattle was significantly lower in the year after the FMD outbreak (2001) compared to the period before the FMD outbreak (2000), but importing cattle also increased in time. The number of animals that were moved for export was significantly higher in the years 2001 and 2004 than in the year 2000: the export level was almost twice as high in 2004 than before the FMD outbreak (2000). The number of animals that were moved off-farm for destruction was significantly higher in the six months after the FMD outbreak (2001) compared to before the FMD outbreak, but decreased in time. No significant differences were found in the number of animals that were moved off-farm for slaughter before and after the FMD outbreak.

**Table 2:** The mean number of cattle movements (*10^**3**^**) per month before FMD (2000) and after FMD (2001–2004) in the Netherlands**

***Cattle movement type***	***Before FMD***	***After FMD***			
	**2000**	**2001**	**2002**	**2003**	**2004**
Off-farm for live trade	226.0^a*^	170.3^b^	193.6^a,b^	200.2^a,b^	201.5^a,b^
	[193.2-263.1]	[136.7-180.4]	[158.5-216.4]	[193.0-209.7]	[172.8-225.5]
Off-farm for slaughter	162.3^a^	151.0^a^	150.1^a^	150.7^a^	159.2^a^
	[143.0-183.5]	[137.7-166.3]	[134.5-165.9]	[127.7-169.3]	[135.9-174.2]
Off-farm for destruction	10.9^a^	17.2^b^	15.6^a,b^	15.9^b^	15.4^a,b^
	[9.2-12.8]	[16.0-20.2]	[14.4-19.4]	[13.5-18.1]	[13.5-18.1]
Off-farm for export	7.7^a^	13.2^b^	11.4^a,b^	11.7^a,b^	14.6^b^
	[6.5-8.9]	[10.5-16.3]	[9.4-16.7]	[8.4-14.9]	[12.5-16.6]
Import	52.9^a^	33.9^b^	43.4^a,b^	47.2^a,b^	44.7^a,b^
	[42.9-61.6]	[22.2-43.5]	[36.0-55.0]	[41.4-59.2]	[26.9-52.3]

The mean number of cattle movements was determined over the months July-December to compare cattle movements across years (i.e. to correct for seasonal effects). The numbers between brackets represent the range [minimum- maximum] of cattle movements (*10^3^). *different letters mean that the numbers between years are significantly different from each other (P ≤ 0.05).

### Cattle movements between and within holding types

Of all the cattle movements off-farm for live trade in the period July 2001-December 2004, most animals were moved off-farm by dairy holdings (48.2%). Despite the small number of holdings (N = 374 in 2004), traders moved 33.7% of all the animals off-farm for live trade. Beef holdings, suckling cow holders, small-scale holdings and young stock raising holdings moved together 18.1% of the animals off-farm for live trade (Table 3).

**Table 3:** Description of on-farm and off-farm movements for different cattle holding types in the Netherlands from July 2001 to December 2004

		**On-farm movements**	**Off-farm movements**				
***Holding type***	***N***	***Percentage closed herds in previous 12 months***	***Percentage of cattle movements off-farm for life trade***	***Percentage farms with contacts in one month***	***Degree (No. of different contact farms in one month)***	***No. Contacts with same farm in one month***	***No. of animals moved/contact in one month***
Traders	374	0	33.7	46.9	13.8	1.2	19.9
					[12.2;14.8]	[1;1.3]	[1.8;29.7]
Young stock raising farms	1,140	0	1.5	57.8	1.5	1.3	2.9
					[1.4;1.6]	[1;1]	[1;3]
Beef herds	3,283	0	8.6	13.3	1.5	1.1	19.9
					[1.5;1.6]	[1;1]	[1;24]
Suckling cow holders	4,624	33.6	4.7	34	1.7	1.1	2.6
					[1.6;1.8]	[1;1]	[1;2.8]
Small-scale farms	15,310	45.5	3.3	12.3	1.3	1.1	2.1
					[1.3;1.3]	[1;1]	[1;2]
Dairy herds	23,544	52.6	48.2	84	1.9	1.6	1.6
					[1.7;1.9]	[1; 2]	[1;1.8]

The numbers between brackets are the 25^th^ percentile and 75^th^ percentile.

**Figure 2:**
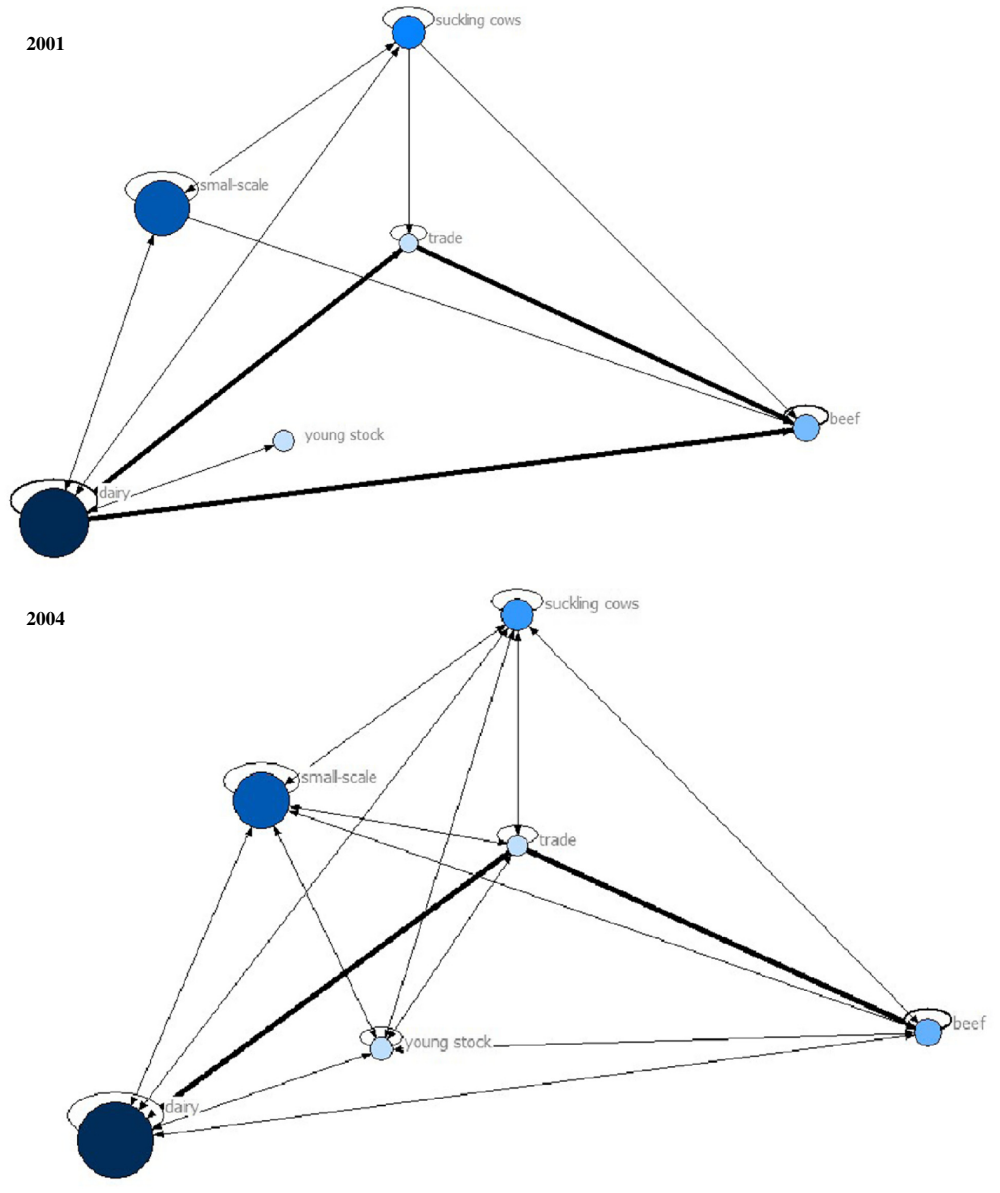
**Contact structure between Dutch cattle holding types in 2001 and 2004.** Contact structure between and within Dutch cattle holding types in July-December 2001 (just after the FMD outbreak) and July-December 2004. The size of the dots represents the number of holdings per holding type. The thicker the lines, the more cattle movements off-farm for live trade (with a minimum of 1,000 movements/month) between or within (loops) holding types.

**Figure 3:**
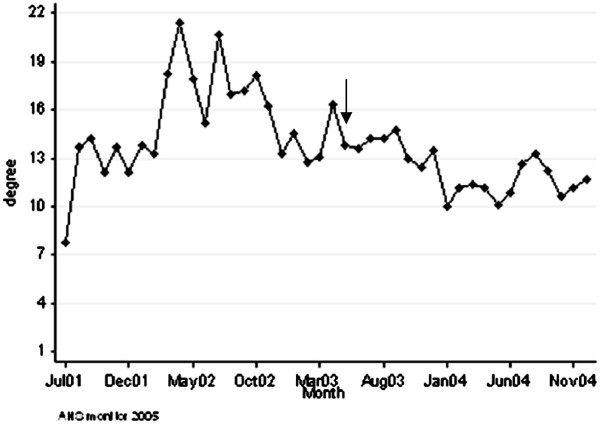
**Mean number of different farms traders have contact with.** Mean number of different holdings traders have contact with by off-farm movements for live trade (degree) in the Netherlands over the period from July 2001 to December 2004. The arrow represents the month in which movement restriction regulations were eased from 30 to 21 days.

A visual comparison was made between the contact structure in 2001 just after the FMD outbreak and the contact structure in 2004 (Figure 2) to explore if the movement restriction regulations caused a durable change in the cattle contact structure between and within holding types. However, our results showed that the change in contact structure just after the FMD outbreak was only temporarily. The main structural changes consisted of a decrease in the number of cattle movements from dairy directly to beef holdings (from an average of 36,052 movements per month in 2001 to 6,331 movements per month in 2004). In addition, the mean number of cattle movements from dairy holdings to traders increased from 40,426 movements per month in 2001 to 74,756 movements per month in 2004. Moreover, the mean number of movements from traders to beef holdings increased from 39,537 movements per month in 2001 to 68,003 movements per month in 2004. Thus, in the period from 2002 to 2004 traders played their central role again in the network. Indeed the mean degree (i.e. number of different holdings an UFI has contact with by off-farm movements) for traders was 8.8 holdings per month in 2001 compared to 16.9 farms per month in 2002, 13.8 holdings per month in 2003 and 11.4 holdings per month in 2004 (Figure 3).

### Network features

#### Closed holding system and holding contacts

**Figure 4:**
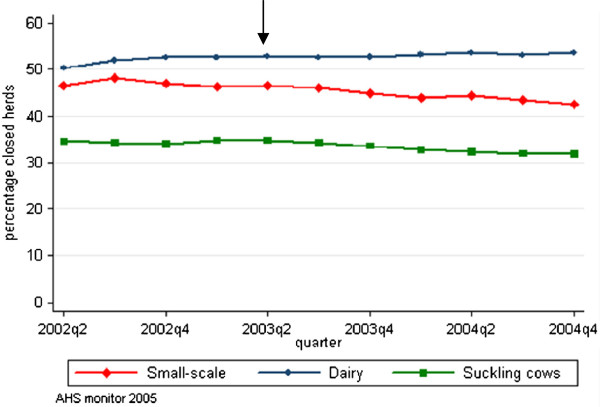
**Mean percentage of closed holdings per holding type.** Mean percentage of closed holdings (no animals moved on-farm in the last 12 months) per holding type in the Netherlands over the period from July 2002 to December 2004. The arrow represents the quarter in which movement restriction regulations were eased from 30 to 21 days.

In the period of July 2002 to December 2004 all traders, beef and young stock raising holdings moved animals on-farm within a 12-month period (i.e. 100% open herds), whereas 52.6% of the dairy holdings were closed and did not move animals on-farm within a 12-month period. In addition, 33.6% of the suckling cow holders and 45.5% of the small-scale holdings were closed herds (Table 3). The proportion of closed holdings was constant in time, except for small-scale holdings. Within this holding type, the percentage of closed herds decreased in time with 5.3% in 2.5 years (Figure 4).

**Figure 5:**
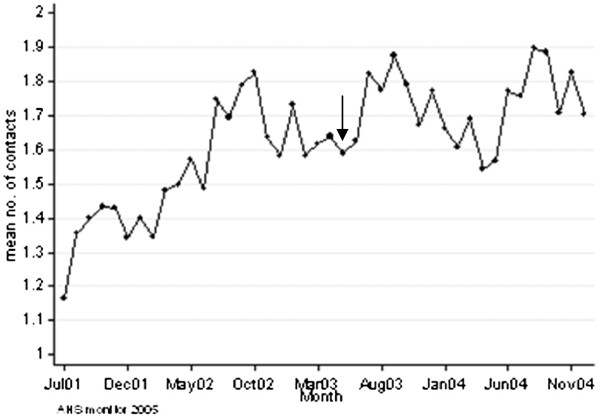
**Mean number of contacts that dairy holdings have with the same holding.** Mean number of contacts that dairy holdings have with the same holding by off-farm movements for live trade in the Netherlands over the period from July 2001 to December 2004. The arrow represents the month in which movement restriction regulations were eased from 30 to 21 days.

Most dairy holdings moved one or more animals off-farm for live trade every month (i.e. 84.0% of the dairy holdings had contacts by off-farm movements), whereas most of the beef holdings and small-scale holdings did not move animals off-farm for live trade every month (Table 3). In addition, the number of different contact holdings by off-farm movements (degree) was highly variable between the holding types: traders had contact with most different holdings (mean of 13.8 different holdings per month) with strong fluctuations in time (Figure 3), whereas the other holding types had far less contacts with different holdings ranging from 1.3 to 1.9 holdings per month, which remained the same in the period after the FMD outbreak (data not shown). Dairy holdings had the highest number of contacts with the same holding, i.e. 1.6 contacts, which was fluctuating within and between years and increased in time (Figure 5). The other holding types had only 1.1 to 1.3 contacts per month with the same holding, which did not change in time (data not shown). The mean number of animals moved per contact was highest for beef holdings and traders: 19.9 animals per contact. For all holding types, the mean number of animals moved per contact remained the same over time (data not shown). For each of the network features, no effect of the ease of movement restriction regulations (since May 2003) was seen.

#### Direct distances

**Figure 6:**
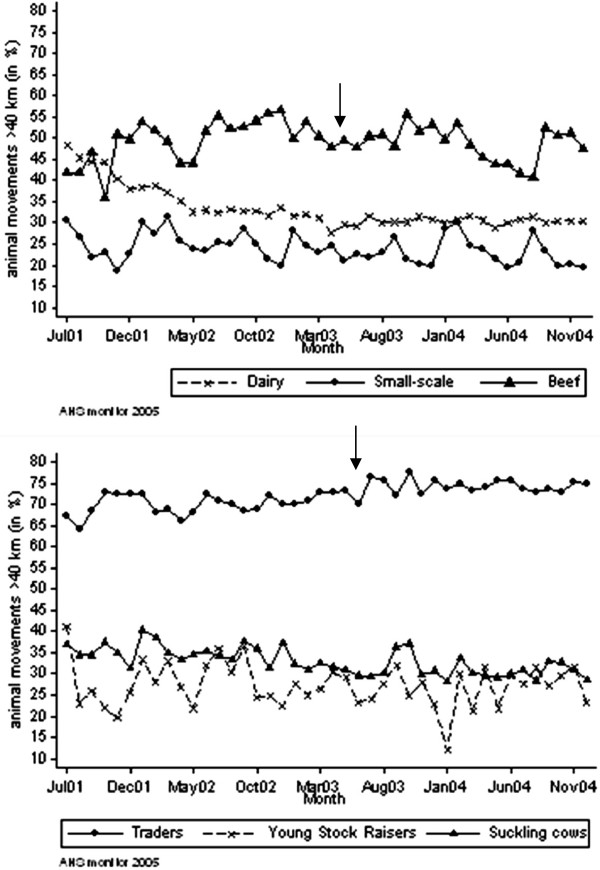
**Percentage of direct distances of cattle movements off-farm for live trade.** Percentage of direct distances of cattle movements off-farm for live trade over a distance of more than 40 km per holding type in the Netherlands over the period from July 2001 to December 2004. The arrow represents the month in which movement restriction regulations were eased from 30 to 21 days.

For 0.6% of the holdings the x and y co-ordinates were missing. Therefore, the distance could not be calculated for 0.4% of the cattle movements off-farm for live trade. The mean direct distance for all movements off-farm for live trade in the period July 2001-December 2004 was highest for traders: 70% of the movements off-farm for live trade were over a distance of more than 40 km. For beef holdings 47% of the movements off-farm for live trade were over a distance of more than 40 km. In case of dairy holdings and suckling cow holders, 32% of the movements off-farm for live trade were over a distance of more than 40 km. Finally, movements off-farm for live trade of small-scale and young stock raising holdings were over a distance of more than 40 km in 23% and 26% of the cases, respectively. Direct distances were steady in time for all holding types and no effect on the ease of movement restriction regulations was seen (Figure 6).

## Discussion

The analyses carried out in this study identified important characteristics and dynamics of cattle movement patterns between and within cattle holdings across years. With these analyses, the efficacy of movement restriction regulations was determined in the short (up to one year) and long (up to 3.5 years) term. Our results acknowledged what has previously been reported by Velthuis and Mourits [11] and showed that trading cattle strongly decreased just after the FMD outbreak (2001). This change was especially seen in the strong decline of cattle movements off-farm for live trade and the decreased import of animals by traders. However, our study also showed a subsequent increase of the number of cattle imports and movements off-farm for live trade in the years 2002–2004 despite retained movement restriction regulations after the 2001 FMD outbreak. This increase was higher than expected as the number of these movements was practically as high as before the FMD outbreak. Traders regained their central role again in the network and the number of traders increased more than two-fold from 160 in 2001 to 374 in 2004. It was assumed that holding owners enforced the rules, because when they violated the rules, a blockade was imposed on their holding. Quantifying the Dutch cattle contact structure between and within holding types up to 3.5 years after the FMD outbreak gave evidence that the post-FMD movement restriction regulations were not able to reduce the number of cattle movements in the longer term. Obviously, the fear of farmers for introduction of FMD and culling of cattle has temporarily changed the cattle contact structure. Similar results were found in the UK [12]. They used a more complex network analysis on holding level to determine the efficacy of movement restriction regulations over time. Our study showed, that using simple network features on holding type level produced the same results. However, in our study design the variation between individual holdings in the movement network and the impact of those cattle holdings on the transmission of diseases is unknown. Other studies have shown that the identity of individual holdings responsible for making network connections can have a significant impact on the infection dynamics [8,16,17]. However, our study focused on network connections between holding types because different holding type authorities exist in the Netherlands that design and enforce type-specific regulations.

The reduced efficacy of the movement restriction regulations can increase transmission of diseases between holdings. Therefore, quantification of the contact structure at any time is essential to assess the compliance with legislation. The effect of the movement restriction regulations in this study was assessed by the change in the contact structure between and within holding types. However, other regulations and economic drive, such as cattle prices, may also have affected the contact structure. However, no evidence exists of a major drive that could have affected the contact structure in the years 2001 to 2004 and therefore the impact on our analyses is considered not serious or even negligible.

As more and more countries hold databases on cattle movements, the analyses that were carried out in this study could also be performed on data concerning cattle movements in other countries. A possible situation may arise that despite movement restriction regulations, the contact structure changes in such a way that the risk of transmission of diseases between holdings increases more than expected and is also present in other countries.

This study also showed that the contact structure between and within Dutch cattle holding types was not random. For example, we found that most dairy holdings had contacts with beef holdings every month. As Dutch dairy holdings have no specific calving season, these movements are due to the off-farm movements of bull calves for beef-rearing and female calves for young stock raising. In addition, despite the small numbers, traders (incl. shows, markets and collection centres) played a central role in the network in the years 2002–2004. Many cattle holdings are connected by movements to and from traders, which are often mixing animals from different holdings. The mixing of animals has been suggested as an important factor in disease dissemination [6]. Moreover, our study showed that movements from traders frequently occurred over long distances. As a result, diseases can easily spread to other areas. In other studies about contact networks between cattle holdings and between swine holdings, a similar non random contact structure was seen [9,10,18]. Data describing contact structures between holding types can be very useful for epidemiological studies on the spread of animal diseases. However, in many mathematical models on disease transmission it is assumed that contacts between and within holding types are random, while in reality this is not the case. This assumption is especially not valid when it comes to crisis management. In most cases only particular individual cattle holdings are an important risk factor in the transmission of diseases [19]. The use of heterogeneous contact parameters has shown to describe animal movement data better than random contact parameters [20]. In addition, Dickey et al. [21] stated that the use of heterogeneous contact parameters predict the transmission of FMDV more accurately than random contact parameters. Moreover, Vernon and Keeling mentioned that due to temporal structures within the dynamic network, static networks consistently fail to capture the predicted epidemic behaviour associated with dynamic networks and therefore recommend to use dynamic networks [22]. Therefore, for efficient disease control it is very important to quantify non-random factors in the contact structure to provide a better understanding of disease transmission. In addition, it allows more realistic comparison of control strategies in case of disease modelling.

Census data from the Dutch Identification and Registration organisation were used to describe the contact structure of the Dutch cattle industry. In the Netherlands, farmers are obliged to report cattle movements. However, compliance is unknown and there can still be some bias in the data due to errors or omissions in the numbers and type of stock moved. In addition, movements of trucks that carry out animal transfers are not available in this database, whereas these trucks could also be an important risk factor for the transmission of diseases. Moreover, other potential transmission routes like the movements of equipment, wildlife and personnel were not included in the I&R database. These biases could underestimate the number of cattle movements between herds of the same and different herd types and with that the risk of disease transmission between cattle herd types. Despite these biases, the database includes detailed information on all cattle movements in the Netherlands over several years. Since there is no evidence that these biases are unevenly dispersed over the years, we think that the biases will probably have a minor impact on our analyses. Acknowledging the limitations, which are also present in other countries like the UK, we believe that the analyses provide useful information on cattle movement patterns in the cattle industry. This knowledge can be important in the early phase of a disease outbreak by providing a better focus on tracing activities [23].

## Conclusions

Quantifying the Dutch cattle contact structure between and within holding types up to 3.5 years after the FMD outbreak gave evidence that the post-FMD movement restriction regulations were not able to reduce the number of cattle movements in the longer term. With that the risk of a large epidemic increased.

The analyses provide useful information on cattle movement patterns, which can be important for targeted disease control and for assessing compliance with legislation. This information can be important in the early phase of a disease outbreak, by providing a better focus on tracing activities and disease modelling. Further research is necessary to determine the variation between individual holdings in the movement network and the impact of those cattle holdings on the transmission of diseases.

## Competing interests

The authors of this manuscript declare that they have no competing interests.

## Authors' contributions

HB carried out the validation and statistical analyses done in this study and drafted the manuscript. CJMB participated in the design and coordination of the study and helped to draft the manuscript. AS has revised the manuscript critically for important intellectual content. GVS participated in the design and coordination of the study, helped to draft the manuscript and revised the manuscript. All authors read and approved the final manuscript.
